# Elemental analysis of contemporary dental materials regarding potential beryllium content

**DOI:** 10.1038/s41598-022-21068-9

**Published:** 2022-11-09

**Authors:** F. Burkhardt, S. Pieralli, T. Bergfeldt, G. Wemken, C. Wesemann, D. Stolz, B. C. Spies

**Affiliations:** 1 Department of Prosthetic Dentistry, Faculty of Medicine, Medical Center, Center for Dental Medicine, University of Freiburg, Freiburg, Germany; 2 Institute of Applied Materials, Applied Material Physics (IAM-AWP), Karlsruhe Institute of Technology (KIT), Eggenstein-Leopoldshafen, Germany; 3 Faculty of Medicine, Medical Center –Clinic of Respiratory Medicine, University of Freiburg, Freiburg, Germany

**Keywords:** Health care, Health occupations, Medical research, Risk factors

## Abstract

Exposure to beryllium (Be) can lead to lung pathologies, such as chronic beryllium disease (CBD). This occupational illness has been more prevalent among dental technicians compared to the non-exposed population. Although most manufacturers state that dental materials are Be-free, this prevalence raises the question of whether the materials are completely devoid of Be-traces. Thus, the objective of the present study was to analyze the elemental composition, with emphasis on Be, of a wide range of commercially available dental materials frequently used by dental laboratories. Samples of 32 different materials were collected and analyzed using inductively coupled plasma-optical emission spectrometry (ICP-OES) and X-ray fluorescence spectroscopy. The results showed that the Be content was below the limit of quantification in all included samples (< 0.00005 mass-%). Therefore, it can be concluded that possible traces of Be were below clinical relevance in dental materials. Exposure of dental technicians to alternative Be sources should be further evaluated.

## Introduction

Beryllium (Be) is a chemical element and naturally occurring lightweight metal that finds industrial application in electronics, aerospace, and defense equipment^1–3^. In the field of dentistry, Be has been used in nickel–chromium^4,5^ alloys with contents of up to 2.05 mass.%^6^ for the fabrication of prosthetic reconstructions. Be reduces the melting temperature, decreases the surface tension, and increases the bond strength between metals and ceramics^7^. Furthermore, it improves the castability and polishing of non-precious alloys.

Manufacturing and processing of Be-containing materials is highly toxic, and workers are exposed to the inhalation of Be particles, fumes, or solutions^8^. Brief exposure can lead to the development of a rare condition called acute berylliosis^9^, while long-term contact can cause Be sensitization (BeS)^10^ and chronic Be disease (CBD), also known as chronic berylliosis^10,11^. An official statement of the American Thoracic Society assessed the prevalence of BeS between 0.9 and 14.6% and of CBD between 0.0 and 7.8%^1^. BeS represents an immunologically-mediated response to the metallic element without evidence of disease, while CBD is considered an incurable occupational lung condition and is often misdiagnosed with sarcoidosis or other granulomatous lung disorder^10^. Symptoms of CBD are cough, dyspnea, fatigue, fever, night sweats, and weight reduction^3,8^ with potential progression to the loss of respiratory function^12^. A history of occupational exposition to Be, positivity to the beryllium lymphocyte proliferation test (BeLPT) and a bioptic examination, confirming a granulomatous inflammation of the lungs, are considered signs for definitive diagnosis of CBD^1^. The incubation periods can last up to three decades^13^. Due to the available evidence of carcinogenicity in humans and the risk of developing lung cancer by occupational exposure, Be and Be compounds have also been classified as category 1 carcinogens by the International Agency for Research on Cancer^14^.

As a consequence of the increased occupational exposure to Be in dental laboratories, dental technicians appear at a higher risk of primarily developing CBD^8,15–18^. Therefore, to protect workers, the Occupational Safety and Health Administration recently established a new limit of 0.2 µg of Be per cubic meter of air for an exposure duration of eight hours or of < 2 µg of Be per cubic meter of air for more than 15 min^18^. According to the current ISO standard for fixed and removable restorations (ISO 22674:2016), the limit value for Be in metallic materials is 0.02% (mass fraction)^19^. Exposure to Be is considered the causal agent for CBD development, and it remains unclear why dental technicians might be more affected. Therefore, the present study aimed to determine the elemental composition of commonly used dental materials and assess the exact amount of Be. Both, non-precious and precious metal alloys used to fabricate prosthetic reconstructions were included. Furthermore, different types of dental ceramics, titanium alloys, polymethyl methacrylate (PMMA), polyether ether ketone (PEEK), and polycarbonate were analyzed. Inductively coupled plasma-optical emission spectrometry (ICP-OES) represents a highly sensitive analytical technique with wide elemental coverage and was applied in the present study^20,21^ The null hypothesis at study conceptualization assumed that evaluated materials contain traces of Be.

## Material and methods

### Study design

The analytical work was performed by the Institute of Applied Materials–Applied Material Physics of the Karlsruhe Institute of Technology (KIT), Eggenstein-Leopoldshafen, Germany. The study proposal for cooperation between the Department of Prosthetic Dentistry and Clinic of Respiratory Medicine of the University of Freiburg, Freiburg, Germany, and the KIT has been approved and accepted by the Karlsruhe Nano Micro Facility in 2020.

### Investigated materials and interdisciplinary cooperation

Four non-precious (Co–Cr) and five precious (Au) alloys were evaluated in the present investigation. In addition, seven ZrO_2_-based ceramics, two feldspathic ceramics, one lithium disilicate glass-ceramic (Li_2_Si_2_O_5_), one nano-fluorapatite glass-ceramic, and one nano-hybrid composite for veneering were included. Furthermore, five implant-supported abutments made of titanium or ZrO_2_, three PMMA-based materials, one polycarbonate, and one PEEK were examined. The evaluated samples represented a selection of the most frequently used materials for each category in two German dental laboratories, which provided the samples for elemental analysis. An overview of the investigated materials and their commercial name and article number is given in Table 1.

**Table 1:** Investigated materials.

Manufacturer	Article no.	Sample name	Material
Kulzer	H-01028	Heraenium EH	Co–Cr alloy
Kulzer	M-01068	Heraenium PW	Co–Cr alloy
Dentaurum	102-620-00	Remanium Star	Co–Cr alloy
Shera	401,070	Sheraheavy-Metal	Co–Cr alloy
Kulzer	SW12002-10	Mainbond EH	Au alloy
Kulzer	SW12004-10	Bio Herador GG	Au alloy
Kulzer	SW12035-0010	Bio Maingold SG	Au alloy
Kulzer	SW12016-10	Heraloy G	Au alloy
Koos Edelmetalle	30,033	Ecobest	Au alloy
Zirkonzahn	ZRAB8001	ICE Zircon Translucent	Oxide ceramic
Kuraray Noritake	6951	Katana™ Zirconia ML	3Y-TZP
Zirkonzahn	ZRFC8021A02	Prettau® 4 Anterior® Dispersive®	5Y-TZP
Ivoclar Vivadent	725,196	IPS e.max ZirCAD Prime	3Y-TZP + 5Y-TZP
Pritidenta	220,841	priti®multidisc ZrO2 Extra Translucent	4Y-TZP
Dental Direkt	G852017	DD cubeX^2^® ML	5Y-TZP
Zirkonzahn	ZRAB8001	ICE Zircon Translucent A3	Oxide ceramic
Ivoclar Viviadent	247,590	IPS e.max Press LT	Lithium disilicate glass-ceramic
Ivoclar Vivadent	168,424	IPS e.max Ceram	Nano-fluorapatite glass-ceramic
GC Europe	622,643	Gradia™ Plus	Nano-hybrid composite
Kuraray Noritake	35,101,010	Ex-3	Feldspathic ceramic
Kuraray Noritake	901,053	Cerabien™ ZR	Feldspathic ceramic
Zirkonzahn	ZRAD8001	Prettau® Zirkon 95H10	Oxide ceramic
Straumann	48,710	RN Variobase	Implant abutment
SIC	935,727	SIC Standard Abutment	Implant abutment
Kulzer	30,503,871	cara i-abutment® titanium	Implant abutment
Zirkonzahn	BSAF0893	Set ZZ Base B-C-ABCH	Implant abutment
Tizian Blank	624,939	Tizian Blank Zirkoniumdioxid	Implant abutment
Organical CAD/CAM	BL00067-3700	Organic PMMA ECO	PMMA
Organical CAD/CAM	N/A	Organic PMMA DISC Multicolour 5-Layer	PMMA
Organical CAD/CAM	N/A	Organic PMMA ECO Clear	PMMA
Ernst Hinrichs Dental	242,774	JUVORA™ PEEK	PEEK
Schütz Dental	220,361	Tizian Blank	Polycarbonate

### Preanalytical sample procession

The samples were divided into seven different groups depending on their different chemical composition. In Table 2 the preanalytical preparations and the chemical digestion are described for each group.

**Table 2:** Preanalytical preparations and chemical digestion for each included group.

Sample name	Cutting^(1)^	Pestle^(2)^	Etching^(3)^	Sample weight^(4)^ mg	Chemical decomposition			Acid mixture						Fill up	
					graphite oven ^(5)^	flux ^(6)^	micro wave ^(7)^	ml						Volume	Solution
					min °C	LiBO_2_/LiBr g °C	min °C	HF suprapure	HCl subb (ml)	HNO_3_ subb (ml)	H_2_O_2_ suprapure	H_2_SO_4_ suprapure	Ultrapure water	ml	
RN Variobase	x	–	x	90–120	120 80	–		1	6	2	–	–	2	50	Ultrapure water
SIC Standard Abutment															
cara i-butment® titanium															
Set ZZ Base B-C-ABCH															
Tizian Blank Zirkoniumdioxide	x	–	–	120–220	–	–	3 / 30 150 / 250	–	–	6	–	4	–	50	Ultrapure water
JUVORA™ PEEK															
Mainbond EH^(8)^	x	–	–	180–240	240 80	–	–	–	12	4	–	–	–	50	10% HCl
Bio Herador GG															
Bio Maingold SG^(8)^															
Heraloy G															
Ecobest^(8)^															
Tizina Blank	x	–	–	88–185	-	-	5/15 150/180	–	–	10	2	–	–	50	Ultrapure water
Organic PMMA ECO															
Organic PMMA DISC Multicolour 5-Layer															
Organic PMMA ECO Clear															
ICE + Farbe	–	x	–	150	–	2/0.02 1000	–	–	HCl 1:1 25	HNO_3_ 1:1 25	–	–	–	100	Ultrapure water
ICE Zircon Transluzent															
KatanaTM Zirconia ML															
Prettau® 4 Anterior® Dispersive®															
IPS e.max ZirCAD Prime															
priti®multidisc ZrO2 Extra Translucent															
DD cubeX^2^® ML															
Sheraheavy-Metal	x	–	–	100–129	720 90	–	–	2	6	2	–	–	2	50	Ultrapure water
Heraenium EH^(8)^															
Heraenium PW^(8)^															
Remanium Star^(8)^															
e.max Press LT	–	x	–	120–150	-	2 / 0.02 1000	–	–	–	HNO_3_ 10% 50	–	–	–	100	Ultrapure water
e.max Ceram															
GradiaTM Plus															
Ex-3															
Cerabien™ ZR															
Prettau® Zirkon 95H10															

(1) cutting edge made of steel (1 BR/6, Peddinghaus, Germany).

(2) mortar mill made of Si3N4 (SRS-2000, Analysen Geräte GmbH, Leutkirch, Germany).

(3) mixed acid (HCl 35% sub., HNO3 subb., ultrapure water (OmniaPure, stakpure GmbH, Niederahr, Germany)).

(4) weighing accuracy ± 0.05 mg; (XP56, Mettler-Toledo, Gießen, Germany).

(5) EasyDigest, Analab, Hoenheim, Germany.

(6) FLUXER F1, Equilab S.A., Madrid, Spain.

(7) Speedwave XPERT, DAK 100, Berghof, Germany.

(8) not completely solved.

### Inductively coupled plasma-optical emission spectrometr

Each sample solution was diluted several times depending on the concentration of the various elements. Instead of using volumetric dilution methods, the sample solution and ultrapure water were weighed (XP 205, Mettler-Toledo, Gießen, Germany). Analysis of the elements was accomplished with four different calibration solutions and an internal standard (Sc) by ICP-OES (iCAP 7600 ICP-OES Duo, Thermo Fisher Scientific Inc., Waltham, MA, USA) (Table 3). For Be the solution was, if necessary, matrix adapted (Ti, Co, Cu, Zr, Mo, Pd, In, W, Pt, Au). The range of the calibration solutions extended from 0.0005 to 0.01 mg/l. One to three wavelengths of Be were used for the calculation.

**Table 3:** Instrument settings for ICP-OES.

ICP	Peristaltic pump	
	Mira Mist peek nebulizer	Gas flow 0.6 (L/min)
	Cyclon teflon spray chamber	
	Ceramic torch with ceramic injector tube	
	RF power (W)	1150
	Auxiliary gas flow	0.5 (L/min) for main compounds
		1.0 (L/min) for high matrix content
Wavelength (nm)	Be	234.861; 313.042; 313.107
	Al	176.638; 308.215; 394.401; 396.152
	Si	212.412; 221.667; 251.611
	K	766.490; 769.896
	Ti	323.452; 334.188; 334.941; 337.280; 338.376
	V	268.796; 292.464; 309.464
	Cr	205.560; 206.157; 267.716
	Co	228.616; 230.786; 238.892
	Cu	213.598; 324.754; 327.396
	Zn	202.548; 206.200; 213.856
	Ga	287.424; 294.364; 403.298; 417.206
	Y	371.030; 377.433; 437.494
	Zr	339.198; 346.823; 348.115; 349.621; 357.685; 383.676
	Mo	202.030; 203.844; 204.598
	Pd	324.270; 340.458; 360.955
	Ag	243.779; 32.068; 338.289
	In	230.606; 303.936; 325.609; 410.172
	Sn	175.790; 189.989; 181.120
	Ba	230.424; 233.527; 413.066
	Hf	251.303; 264.141; 277.336
	W	207.911; 220.448; 224.875
	Pt	177.709; 203.646; 214.423; 224.552
	Au	197.819; 208.209; 211.068; 242.795; 267.595

### X-ray fluorescence spectroscopy

All samples were analyzed semiquantitative via X-ray fluorescence spectroscopy (XRF) (Pioneer S4, Bruker AXS, Karlsruhe, Germany) against different universal calibrations depending on the material of the samples (metal, oxide, etc.).

### Quality control

The certified ICP calibration solutions (Aesar, Thermo Fisher (Kandel) GmbH, Karlsruhe, Germany, CPAChem, Bogomilovo, Bulgaria) were controlled with another certified ICP solution from a different producer (Agilent, Waldbronn, Germany; Merck, Darmstadt, Germany). The recovery of these standards in matrix-adapted solutions was between 95 and 105%.

### Descriptive statistics

Results of the elemental analysis are described in Tables 2, 3, 4, 5 and 6 as the mean outcome, standard deviation (SD) and measurement uncertainty ( ±). Data regarding the oxides are semiquantitative results determined with XRF against a universal calibration. The results were normalized to 100.

**Table 4:** ICP-OES results of non-precious metal alloys.

Element	Unit	LOQ	Heraenium EH			Heraenium PW			Remanium Star			Sheraheavy-Metal		
			Mean	SD	±	Mean	SD	±	Mean	SD	±	Mean	SD	** ± **
Be	mass-%	0.00001	< 0.00001	–	–	< 0.00001	–	–	< 0.00001	–	–	< 0.00001	–	–
Cr	mass-%	0.1	26.9	–	2.7	23.0	–	2.3	23.0		1.2	30.9	0.1	1.5
Mn	mass-%	0.100	0.568	–	0.114	0.677	–	0.169	< 0.100	–	-	0.515		0.103
Fe	mass-%	0.050	0.012	–	0.003	4.160	–	0.416	0.034	–	0.009	0.564	–	0.056
Co	mass-%	0.1	63.4	–	6.3	54.7	–	5.5	59.8	4.6	3,0	59.1	0.2	3.0
Ga	mass-%	0.37	< 0.37	–	–	< 0.37	–	–	< 0.37	–	–	< 0.37	–	–
Mo	mass-%	0.09	7.57	–	1.14	< 0.09		–	0.22		–	4.97	0.01	0.25
In	mass-%	0.06	< 0.06	–	–	< 0.06		–	< 0.06		–	< 0.06		–
Sn	mass-%	0.03	< 0.03	–	–	< 0.03		–	< 0.03		–	< 0.03		–
W	mass-%	0.07	< 0.07	–	–	15.90	-	1,59	9.56		–	< 0.07	–	–
Pt	mass-%	0.05	< 0.05	–	–	< 0.05		–	< 0.05		–	< 0.05	–	–
Au	mass-%	0.05	< 0.05	–	–	< 0.05		–	< 0.05		–	< 0.05	–	–
Total	mass-%		98.44989			98.43700			92.61400			96.04884		

*LOQ* limit of quantitation, *SD* standard deviation, ± : measurement uncertainty.

**Table 5:** ICP-OES results of precious metal alloys.

Element	Unit	LOQ	Mainbond EH			Bio Herador GG			Bio Maingold SG			Heraloy G			Ecobest		
			Mean	SD	±	Mean	SD	±	Mean	SD	±	Mean	SD	±	Mean	SD	±
Be	mass-%	0.000004	< 0.000004	–	–	< 0.000004	–	–	< 0.000004	–	–	< 0.000004	–	–	< 0.000004	–	–
Fe	mass-%	0.200	< 0.200	–	–	0.983	–	0.246	< 0.200	–	–	0.318	–	0.080	0.230	–	0.058
Cu	mass-%	0.53	7.43	0.03	0.37	< 0.53	–	–	12.1	0.1	0.6	< 0.53	–	–	< 0.53	–	–
Zn	mass-%	0.100	0.482	0.003	0.024	< 0.100	–	–	0.473	0.006	0.024	< 0.100	–	–	1.36	0.01	0.07
Ga	mass-%	0.13	n.a	–	–	< 0.13	–	–	n.a	–	–	1.97	0.02	0.10	n.a	–	–
Pd	mass-%	0.40	< 0.40	–	–	< 0.40	–	–	< 0.40	–	–	35.9	0.1	3.6	9.73	0.10	0.49
Ag	mass-%	0.30	15.2	–	1.52	n.a	–	–	14.3	–	1.43	n.a	–	–	29.1	–	2.9
In	mass-%	0.30	< 0.30	–	–	1.65	0.01	0.08	< 0.30	–	–	8.21	0.11	0.41	3.96	0.01	0.20
Pt	mass-%	0.12	8.36	0.04	3.59	11.2	0.1	3.59	3.85	0.01	3.59	< 0.12	-	-	< 0.12	–	–
Au	mass-%	0.2	69.0	0.5	4.2	84.6	0.8	5.2	70.2	0.5	4.3	50.8	0.5	3.1	56.3	0.3	3.4
Total	mass-%		100.472			98.433			100.923			97.198			100.680		

*LOQ* limit of quantitation, *SD* standard deviation, ± : measurement uncertainty; *n.a.* not available.

**Table 6:** ICP-OES results of oxide ceramics.

Element	Unit	LOQ	ICE Zircon Transluzent			Katana Zirconia ML			Prettau® 4 Anterior® Dispersive®			IPS e.max ZirCAD Prime			priti®multidisc ZrO2 Extra Translucent			DD cubeX^2^® ML			ICE Zircon Transluzent A3		
			Mean	SD	±	Mean	SD	±	Mean	SD	±	Mean	SD	±	Mean	SD	±	Mean	SD	±	Mean	SD	±
Be	mass-%	0.00005	< 0.00005	–	–	< 0.00005	–	–	< 0.00005	–	–	< 0.00005	–	–	< 0.00005	–	–	< 0.00005	–	–	< 0.00005	–	–
Y	mass-%	0.01	4.29	0.03	0.21	5.58	0.13	0.28	6.49	0.03	0.32	5.38	0.05	0.27	5.24	0.02	0.26	7.56	0.13	0.38	4.25	0.08	0.21
Zr	mass-%	2.6	68.0	0.6	3.4	67.3	1.5	3.4	66.1	0.3	3.3	67.5	0.8	3.4	67.3	0.3	3.4	65.3	1.2	3.3	67.9	1.3	3.4
Hf	mass-%	0.02	1.49	0.01	0.07	1.29	0.03	0.06	1.29	0.01	0.06	1.50	0.02	0.08	1.45	0.01	0.07	1.42	0.03	0.07	1.55	0.03	0.08
Total	mass-%		73.78			74.17			73.88			74.38			73.99			74.28			73.70		

*LOQ* limit of quantitation, *SD* standard deviation, ± : measurement uncertainty.

## Results

Detailed results of the ICP-OES elemental analysis are shown in Tables 4, 5, 6, 7 and 8.

**Table 7:** ICP-OES results of further investigated ceramic materials.

Element	Unit	LOQ	IPS e.max Press LT			IPS e.max Ceram			GradiaTM Plus			Ex-3			Cerabien™ ZR			Prettau® Zirkon 95H10		
			Mean	SD	±	Mean	SD	±	Mean	SD	±	Mean	SD	±	Mean	SD	±	MW	SD	±
Be	mass-%	0.00002	< 0.00002	–	–	< 0.00002	–	–	< 0.00002	–	–	< 0.00002	–	–	< 0.00002	–	–	< 0.00002	–	–
Na_2_O	mass-%	–	0.42	–	–	3.69	–	–	0.21	–	–	4.71	–	–	4.15	–	–	-	–	–
MgO	mass-%	–	0.26	–	–	-	–	–		–	–	0.32	–	–	0.30	–	–	-	–	–
Al_2_O_3_	mass-%	–	2.22	–	–	8.08	–	–	6.89	–	–	15.9	–	–	12.6	–	–	0.6	–	–
SiO_2_	mass-%	–	76.0	–	–	49.1	–	–	49.8	–	–	64.9	–	–	71.70	–	–	5.41	–	–
P_2_O_5_	mass-%	–	1.98	–	–	0.12	–	–	0.03	–	–	-	–	–	-	–	–	-	–	–
SO_3_	mass-%	–	0.06	–	–	0.05	–	–	0.74	–	–	0.04	–	–	0.03	–	–	–	–	–
K_2_O	mass-%	–	7.53	–	–	7.67	–	–	0.16	–	–	11.0	–	–	7.81	–	–	0.24	–	–
CaO	mass-%	–	0.06	–	–	1.99	–	–	0.13	–	–	0.80	–	–	0.75	–	–	0.06	–	–
TiO_2_	mass-%	–	–	–	–	1.41	–	–	0.30	-	-	-	-	-	-	-	-	0.05	–	–
Cr_2_O_3_	mass-%	-	0.12	–	–	0.04	–	–	–	–	–	0.07	–	–	0.08	–	–	–	–	–
MnO	mass-%	–	–	–	–	–	–	–	–	–	–	–	–	–	–	–	–	–	–	–
Fe_2_O_3_	mass-%	–	0.26	–	–	0.09	–	–	0.09	–	–	0.15	–	–	0.15	–	–	0.26	–	–
Co_2_O_3_	mass-%	–	–	–	–	–	–	–	–	–	–	–	–	–	–	–	–	–	–	–
NiO	mass-%	–	–	–	–	–	–	–	–	–	–	-	–	–	–	–	–	–	–	–
CuO	mass-%	–	–	–	–	–	–	–	–	–	–	-	–	–	–	–	–	0.03	–	–
ZnO	mass-%	–	4.67	–	–	5.01	–	–	–	–	–	0.22	-	-	0.06	–	–	0.06	–	–
SrO	mass-%	–	2.70	–	–	8.37	–	–	0.35	–	–	-	-	-		–	–	–	–	–
Y_2_O_3_	mass-%	–	-	–	–	0.53	–	–	–	–	–	0.10	-	-	0.15	–	–	5.03	–	–
ZrO_2_	mass-%	–	1.40	–	–	9.49	–	–	–	–	–	0.64	-	-	0.56	–	–	85.80	–	–
Nb_2_O_5_	mass-%	–	–	–	–	–	–	–	–	–	–	–	–	–	–	–	–	0.71	–	–
SnO	mass-%	–	–	–	–	3.78	–	–	–	–	–	–	–	–	–	–	–	–	–	–
Sb_2_O_3_	mass-%	–	–	–	–	–	–	–	–	–	–	1.15	–	–	1.24	–	–	–	–	–
BaO	mass-%	–	–	–	–	–	–	–	41.3	–	–	–	–	–	–	–	–	–	–	–
La_2_O_3_	mass-%	–	0.50	–	–	–	–	–	–	–	–	–	–	–	–	–	–	–	–	–
CeO_2_	mass-%	–	1.00	–	–	0.58	–	–	–	–	–	–	–	–	0.42	–	–	–	–	–
Tb_2_O_3_	mass-%	–	0.82	–	–	–	–	–	–	–	–	–	–	–	–	–	–	–	–	–
HfO_2_	mass-%	–	–	–	–	–	–	–	–	–	–	–	–	–	–	–	–	1.80	–	–
Total	mass-%		100.00	100.00	100.00	100.00	100.00	100.00												

*LOQ* limit of quantitation, *SD* standard deviation, ± : measurement uncertainty.

**Table 8:** ICP-OES results of metallic and non-metallic implant abutment materials.

Element	Unit	LOQ	RN Variobase			SIC Standard Abutment			cara i-abutment® titanium			Set ZZ Base B-C-ABCH			Tizian Blank Zirkoniumdioxid		
			Mean	SD	±	Mean	SD	±	Mean	SD	±	Mean	SD	±	Mean	SD	±
Be	mass-%	0.000004	< 0.000004	–	–	< 0.000004	–	–	< 0.000004	–	–	< 0.000004	–	–	< 0.000004	–	–
Al	mass-%	0.04	< 0.04	–	–	5.74	0.08	0.15	5.88	0.11	0.16	5.62	0.10	0.15	n.a	–	–
Ti	mass-%	0.2	100.0	0.5	2.4	89.8	0.1	2.2	89.9	0.1	2.2	90.2	0.1	2.2	11.3	0.2	2.3
V	mass-%	0.13	< 0.13	–	–	4.06	0.02	0.09	3.97	0.03	0.08	3.84	0.02	0.08	n.a	-	-
Total	mass-%		100.0			99.60			99.75			99.66			11.30		

*LOQ* limit of quantitation, *SD* standard deviation, ± : measurement uncertainty, *n.a.* not available.

### Non-precious metal alloys

The limit of quantitation of Be in ICP-OES analysis is 0.1 mg/kg (Table 4). This Be level could not be measured in any non-precious dental alloy sample. Co was the main component in all the samples studied, followed by Cr. Heraenium PW (15.9 mass %) and Remanium Star (9.6 mass %) are the only ones containing W; Heranium PW showed the highest amount of Fe (4.2 mass %).

### Precious metal alloys

The main component of all precious alloys was Au (50.8–84.6 mass-%; Table 5). Maingold EH and Bio Maingold SG presented also Cu, Ag and Pt, whereas in Bio Herador GG the second main element besides Au was Pt. Heraloy G showed the highest amount of Pd (35.9 mass-%) and Ecobest the highest amount of Ag (29.1 mass-%). Be was not determined in any of the precious metal alloy samples tested (limit of quantitation of 0.04 mg/kg).

### Oxide ceramics

Data of the three most present elements are semiquantitative and determined with XRF against a universal calibration. The concentration data can vary from more than 25 to < 5%. The results were normalized to 100. Be concentration of the tested oxide ceramics resulted below the measuring limit of 0.5 mg/kg (Table 6). All the oxide ceramics investigated had a Zr content between 66.1 and 68 mass%. Y was contained in all samples and DD cubeX^2^® ML (7.6 mass-%) showed the highest content. Furthermore, Hf, which belongs to the group of heavy metals, was detected in all samples ranging between 1.29 and 1.55 mass %.

### Other ceramics

As for the oxide ceramics, semiquantitative results were obtained with XRF against a universal calibration. The concentration data can vary by more than 100% at concentrations < 5% and the results were normalized to 100. None of the evaluated samples contained Be (limit of quantitation < 0.2 mg/kg) (Table 7). IPS e.max Press contained 76 mass-% SiO_2_, while IPS e.max Ceram contained 49.1 mass-% SiO_2_ with a higher content of ZrO_2_ (9.49 mass-%) compared to the other groups. Gradia™ Plus is the only investigated nano-hybrid composite containing BaO (41.3 mass-%), the second-largest component in this material after SiO_2_ (49.8 mass-%).

### Implant abutments

The elemental analysis of the implant abutments showed that the SIC standard abutment and the cara i-abutment ® titanium had a similar elemental composition (Table 8). Both consisted of approximately 90 mass-% Ti, 4 mass-% V and 6 mass-% Al. The RN Variobase abutment was measured to be 100 mass-% Ti (with a standard deviation of 0.5 mass-% and measurement uncertainty of 2.4 mass-%). The values for Al, Ti and V were below the respective detection limit. Therefore, a possible Be content was below the detection limit for all the evaluated samples.

### PMMA, PEEK, polycarbonate

The analysis of the three PMMA, one PEEK, and one polycarbonate material revealed a Be content < 0.08 mg/kg for all the evaluated samples (Table 9).

**Table 9:** ICP-OES results of included PEEK-, PMMA- and polycarbonate-based materials.

			Juvora PEEK			Tizian Blank			Organic PMMA Eco A3			Organic PMMA DISC Multicolor 5-Layer			Organic PMMA Eco Clear		
Element	Unit	LOQ	Mean	SD	±	Mean	SD	±	Mean	SD	±	MW	SD	±	MW	SD	±
Be	mg/kg	0.04	< 0.04		–	< 0.08		–	< 0.08		–	< 0.08		–	< 0.08		–

*LOQ* limit of quantitation, *SD* standard deviation, ± : measurement uncertainty.

## Discussion

The objective of this study was to investigate multiple currently used dental materials concerning their possible Be content. To our best knowledge, no studies have conducted an elemental analysis for the detection of Be using a comparable broad spectrum of different dental materials, which includes precious and non-precious alloys, ceramics, PMMA, PEEK, and polycarbonate. Importantly, due to the low occupational exposure limits (0.2 mg Be/m^3^ air), a very sensitive methodology is necessary to determine the concentration of Be at ultra-trace levels.

Be has been widely used in the past decades to manufacture dental appliances^22,23^. To date, Be-exposure is considered "a modern industrial hazard"^24^ which can lead to sensitization and CBD, chronic lung disease^2^. A key factor for the management of CBD is the prevention of workplace-related and environmental Be exposure^25^. Frye et al. described a cluster of workers in an industry not directly related to Be processing and suffering from BeS caused by the high levels of Be contained in the concrete dust^25^. Appropriate protective equipment and preventive measures are mandatory to reduce the risk of respiratory diseases. In addition, routine medical examinations should be provided as for other high-exposure worker categories. Although exposure to Be in working places is being strictly regulated by the Occupation Health and Safety Administration, controlling is difficult^26^. Dental technicians are at higher risk of developing occupational respiratory disorders such as pneumoconiosis, caused by exposure to dust while handling dental materials^27–30^. They still seem to represent a population at higher risk of Be-associated disorders as compared to non-exposed workers despite the increasingly use of Be-free materials^17,31^. Furthermore, while the term "beryllium free" is used by several manufacturers to name their dental products, the concentration threshold for defining a material "free" from Be is still not defined. Further research groups aimed to assess the amount of Be contained in dental materials. Alkmin et al. investigated the microstructural characteristics of eight Ni–Cr alloys in the commerce^6^. The samples were analyzed using an inductively coupled plasma spectrometer (ICP-OES). Of the eight investigated alloys, five presented Be traces up to 2.05 mass-% and in two of these cases, the Be amount was not reported by the manufacturer.

There are different methods for ICP elemental analysis. On one side, inductively coupled plasma mass spectrometry relies on a high-temperature ionization source paired with a mass spectrometer. After nebulization, the samples are atomized, and ions are generated for the mass analysis^20,21^. On the other side, ICP-OES technology is based on the light transmission at specific wavelengths by atoms that move to a lower energy level. Element type and concentration are calculated based on the position and the intensity of the photon rays. All the analytical investigations of this study were performed with ICP-OES, which allows for precise multi-element tracing with high sensitivity and low detection limits.

Within the limit of quantification of the adapted methodic, Be traces ranged from below 0.000004 to 0.00005 mass-% depending on the group of materials analyzed. Based on these analyses, conducted at the ultra-trace level, it can be assessed that traces of Be are not of clinical significance in the evaluated samples. Therefore, the null hypothesis of the present investigation, assuming traces of Be are contained in the investigated dental materials, has to be rejected. Be was not found in the investigated materials, but further independent studies should address the elemental composition of used dental materials, focusing on heavy metals. A thorough understanding of health risks and the development of strategies to minimize occupational exposure to hazards should be continually pursued.

These results, however, raise further questions regarding the increased prevalence of Be-associated disorders in dental technicians and an evidence-based explanation. Firstly, some of the studies were conducted several years ago^8,15,32^, and the identification of health hazards, as well as the consequent restrictions adopted, might have caused the modification of the material compositions by the manufacturers. Secondly, despite the large-scale screening, the analyzed samples represent only a minimal fraction of the materials currently used in dental laboratories. Finally, it should also be considered that this study included only materials used in German dental laboratories, while most recent articles describing the prevalence of Be-associated diseases in dental technicians were assessed in other countries^17,31,33^. Despite the analyses of a large amount of samples by several sensitive methods, this study has a few limitations, including the restriction of the geographic area to Germany and to certain types of material. Analogue evaluations should be considered in future investigations involving a broader group of materials and different countries.

## Conclusions

Based on the described elemental analysis, the following conclusions can be drawn:

- The applied ICP-OES method allowed for a highly sensitive elemental analysis at ultra-trace levels.
- Be concentration was below the respective limit of quantification (< 0.00005 mass-%) for all the evaluated samples.
- Further studies are needed to assess the Be amount in currently commercialized dental materials.

## Data Availability

All data generated or analysed during this study are included in this published article.

## Abbreviations

CBD: Chronic Beryllium disease
BeLPT: Beryllium lymphocyte proliferation test
3Y-TZP: 3 Yttria-stabilized tetragonal zirconia polycrystal
4Y-TZP: 4 Yttria-stabilized tetragonal zirconia polycrystal
5Y-TZP: 5 Yttria-stabilized tetragonal zirconia polycrystal
BeS: Be sensitization
ICP: Inductively coupled plasma
ICP-OES: Inductively coupled plasma-optical emission spectrometry
PEEK: Polyether ether ketone
PMMA: Polymethyl methacrylate
XRF: X-ray fluorescence spectroscopy
